# Stacked-Cup Carbon Nanotube Flexible Paper Based on Soy Lecithin and Natural Rubber

**DOI:** 10.3390/nano9060824

**Published:** 2019-05-31

**Authors:** Amirreza Shayganpour, Sara Naderizadeh, Silvia Grasselli, Annalisa Malchiodi, Ilker S. Bayer

**Affiliations:** 1 Smart Materials, Istituto Italiano di Tecnologia, Via Morego 30, 16163 Genova, Italy; Amirreza.Shayganpour@iit.it; 2 Dipartimento di Informatica Bioingegneria, Robotica e Ingegneria dei Sistemi (DIBRIS), Universita Degli Studi di Genova, Via All’Opera Pia 13, 16145 Genova, Italy; sara.naderizadeh@iit.it; 3 GEA Mechanical Equipment Italia S.p.A., Via da Erba Edoari Mario, 29, 43123 Parma, Italy; Silvia.Grasselli@gea.com (S.G.); Annalisa.Malchiodi@gea.com (A.M.)

**Keywords:** soy lecithin, carbon nanotubes, carbon nanofibers, natural rubber, biocomposites, conductive paper, thermal conductivity

## Abstract

Stacked-cup carbon nanotubes (SCCNTs) are generally referred to as carbon nanofibers (CNFs). SCCNTs are much less expensive to fabricate and are regarded as good polymer modifiers suitable for large-scale production. Flexible, SCCNT-based soy lecithin biocomposites were fabricated using liquid natural rubber latex as binder. Natural polymers and the SCCNTs were dispersed in a green solvent using a benchtop high-pressure homogenizer. The inks were simply brush-on painted onto cellulose fiber networks and compacted by a hydraulic press so as to transform into conductive paper-like form. The resulting flexible SCCNT papers demonstrated excellent resistance against severe folding and bending tests, with volume resistivity of about 85 Ω·cm at 20 wt % SCCNT loading. The solvent enabled formation of hydrogen bonding between natural rubber and soy lecithin. Thermomechanical measurements indicated that the biocomposites have good stability below and above glass transition points. Moreover, the SCCNT biocomposites had high through-plane thermal conductivity of 5 W/mK and 2000 kJ/m^3^K volumetric heat capacity, ideal for thermal interface heat transfer applications.

## 1. Introduction

Flexible nano-carbon-based paper structures have been a very popular research area due to several high-value application potentials, such as components for Li-ion batteries and energy storage devices [1,2,3], supercapacitors [4,5,6], thermoelectric devices [7], electromagnetic shielding [8,9,10], and sensors [11]. Similarly, carbon nanofiber (CNF)-based papers have been developed and studied in detail for nearly identical applications [12,13,14,15]. Electronic devices on paper or conductive paper substrates are ideal for thin and compact electronics that require flexibility and conductivity under multiple folding events with additional biodegradable and compostable properties. Building functional nano-carbon paper substrates is considered to be a greener alternative to others made of plastics and metals [16]. 

In relation to this, electronic waste or e-waste has been a steadily mounting problem in today’s waste management and recycling [17]. Even post-processing and recycling of conventional electronic parts can be dangerous and lead to adverse human health effects [18] and environmental pollution [19]. In addition to various heavy metals, e-waste generates a large amount of plastic waste [20] that could be more complicated to recycle compared to plastic waste originating from packaging, including food packaging [21]. Hence, it is getting increasingly important to find ways to incorporate more carbon-based conductors and biodegradable plastics and resources in consumer electronics without sacrificing performance [22,23]. At the same time, the global biocomposites market size is estimated to reach $46.3 billion by 2025, based on a report published by Grand View Research, Inc. [24]. This is driving the technology of biocomposites in applications like electronics [25,26,27,28]. 

Bio-(nano)composites can be manufactured in various different ways, including extrusion [28,29], emulsions [30], thermoforming [31], fiber impregnation [32], electrospinning [33], and foaming [34], to name a few. In this work, we produced stacked-cup carbon nanotube (SCCNT)–biopolymer inks in a green solvent known as methyl isobutyl ketone (MIBK). MIBK is known to biodegrade readily in the environment [35,36,37]. The inks were then brush-painted on unsized papers (cellulose fiber mats) and thermoformed into a carbon nanotube paper by hot pressing. As binders, we have used soy lecithin and natural rubber gum. Phospholipids from soybeans known as lecithin are permitted as food emulsifiers, and they are also extensively used as emulsifiers in the pharmaceutical, cosmetic, and paint industries [38,39]. Biocomposites containing significant amounts of soy protein or lecithin are very rare in literature [40,41,42]. Lecithin has been successfully used as binder in nanostructured carbon electrodes for laccase-catalyzed oxygen reduction without added mediators for biosensors and biofuel cells. [43]. Natural rubber, on the other hand, has been extensively used in making nanocomposites with carbon nanotubes and carbon nanofibers [44,45,46,47]. It is also a common elastomer to produce stretchable electronic conductors [48].

**Figure 1:**
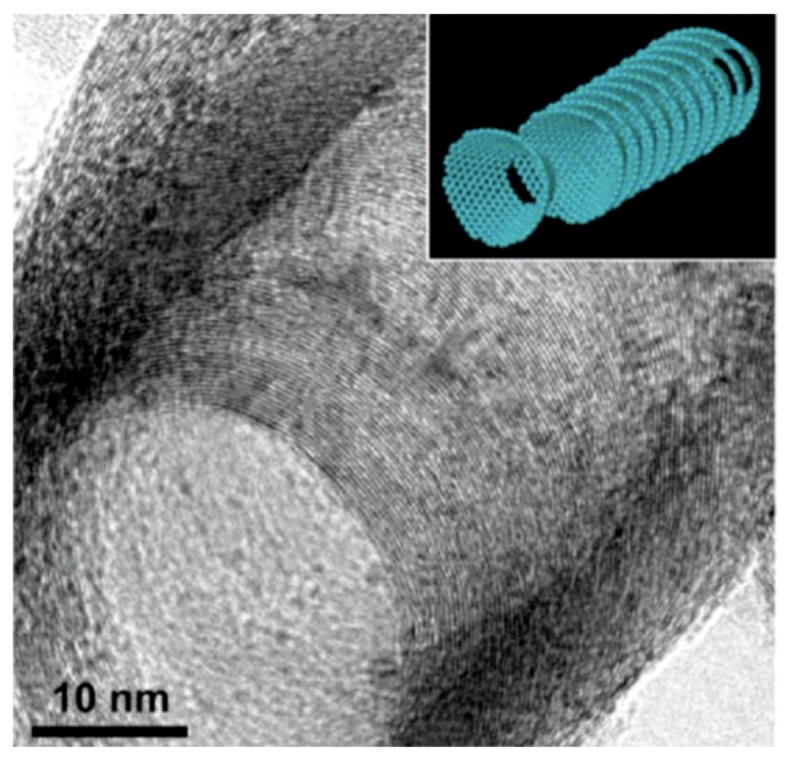
High resolution transmission electron microscope image of a milled stacked-cup carbon nanotube (SCCNT) taken at a tilted angle. Stacked cup morphology is very uniform for every cup. The inset demonstrates a schematic structural model. Reproduced from [49] with permission from American Chemical Society, 2019.

Finally, SCCNTs are a relatively new form of carbon nanostructures that differ significantly in morphology compared to other carbon nanostructures. For instance, conventional single-wall carbon nanotubes are made up of seamless cylinders of hexagonal carbon networks, whereas SCCNTs provide a hollow and tubular morphology, as demonstrated in Figure 1 [49]. They are also known as carbon nanofibers (CNFs) [49,50]. 

Their truncated-cone morphology enables exposure of many reactive edges in the outer and inner surfaces of the hollow tubes. These inner/outer edges can be chemically functionalized, which translates into easier surface modifications that are considered ideal for electronic and catalytic applications [51].

## 2. Materials and Methods 

**Figure 2:**
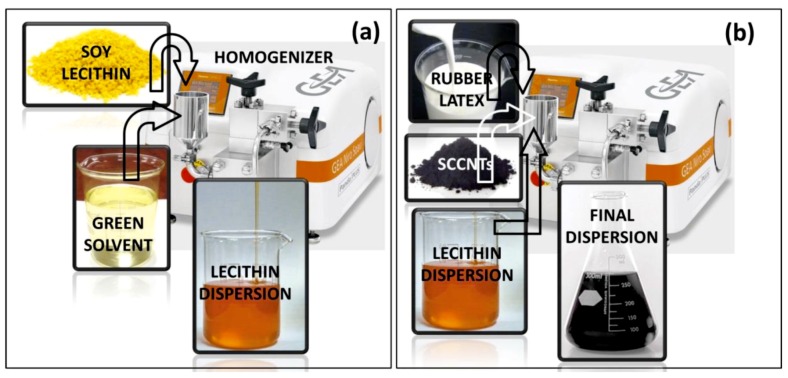
Schematic representation of high-pressure homogenization of soy lecithin in methyl isobutyl ketone (MIBK) (**a**) and blending of lecithin dispersion with SCCNTs and natural rubber (**b**).

The 4-Methyl-2-pentanone (MIBK, ACS reagent, ≥98.5%) was purchased from Sigma-Aldrich (Munich, Germany) and used as received. Liquid natural rubber gum was purchased from Marabu-Creative (Tamm, Germany). The product name is Fixogum. It is 37 wt % natural rubber latex in liquid/solvent. Before use, the liquid content was dried/evaporated under ambient conditions, and the polymer was re-dispersed in MIBK. Soy lecithin was purchased from a local supermarket. The product is called Lecinova, produced by Nutrition & Santé Italia S.p.A. (Milan, Italy). It was used as received. SCCNTs (PR-19-XT-PS) were purchased from Pyrograf Products Inc. (Cedarville, OH, USA). The tubes or fibers were produced in the form of pyrolytically stripped carbon tubes that remove polyaromatic hydrocarbons from tube surfaces. The average nanotube diameter is reported to be 150 nm with a surface area of 25 m^2^/g. As a biodegradable fiber support, cellulosic tissues (Kimwipes™, Kimberly-Clark) were used. The tissues are made up of single ply 100% virgin wood fibers, with no sizing agents or chemicals. Soy lecithin particles were ground into a fine powder using a standard coffee grinder. The powder was added to MIBK slowly, such that a 15 wt % solution was formed. The solution maintained in a closed glass beaker was placed on a magnetic stirrer and stirred for one hour at 60 °C. After cooling to room temperature, the solution was subjected to high-pressure homogenization (GEA Lab Homogenizer PandaPLUS 1000) at 750 bar (10,000 psi) for 5 cycles to produce the final dispersion. The homogenizer is able to process a minimum of 100 mL dispersion. The process is schematically illustrated in Figure 2a. 

**Figure 3:**
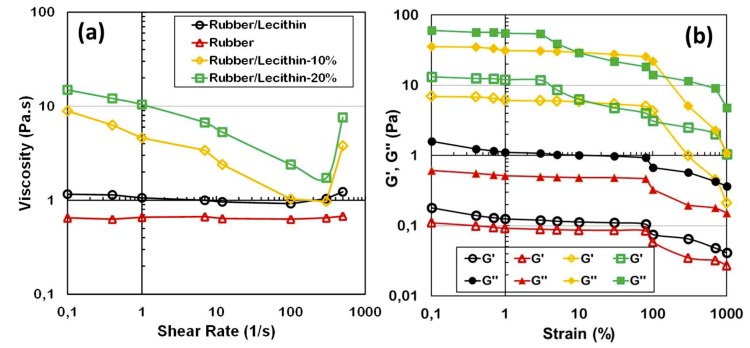
(**a**) Shear rate versus viscosity of selected rubber/lecithin blends and SCCNT-dispersed solutions in MIBK. (**b**) Dynamic rheological response of the same rubber, rubber/lecithin, and SCCNT-dispersed rubber/lecithin suspensions. Note that rubber/lecithin ratio is always kept at 1:1 regardless of the SCCNT concentration. In the legend, for instance, Rubber/Lechitin-10% refers to SCCNT concentration in the polymer blend matrix.

SCCNTs were then added to the high-pressure homogenized lecithin solution by magnetic stirring under room temperature for one hour. The mixture was then placed in an ultrasonic processing water bath (Bransonic^®^ Ultrasonic M) for 2 h at 40 °C. Afterwards, the SCCNT-lecithin mixture was blended with the natural rubber solution in MIBK. The blending was carried out such that in the final solution, the ratio between lecithin and natural rubber was always maintained at 1:1 ratio. Polymer (lecithin + rubber) to SCCNT weight ratio was tuned from 0.05 to 0.35. The final blend was subjected to high-pressure homogenization at 820 bar for 5 cycles and another 5 cycles after pausing for 30 min. The process is schematically illustrated in Figure 3b. For instance, a typical 30 mL SCCNT-dispersed rubber/lecithin solution contained 1 g natural rubber, 1 g lecithin, and 0.4 g SCCNTs. This translates into 17 wt % SCCNT in a dry biocomposite film. Note that the rubber/lecithin ratio of 1:1 has been maintained constant throughout the work.

Viscous characteristics of the inks were measured by steady and dynamic shear experiments using a TA Instruments Discovery Hybrid 2 rheometer with 40 mm parallel plates at room temperature with a gap spacing of 1 mm. Strain amplitude was varied from from 0.1 to 1000% at a frequency of 1 rad/s. Flow sweep experiments were done within a shear rate of 0.1–1000 s^−1^. A pre-shear step (1 s^−1^) was applied for about a minute’s duration before flow sweep measurements. All measurements were repeated three times using different batches to ensure no extreme experimental variations were observed. In general, batch-to-batch viscosity measurement difference was within 15%. 

The microstructure of the fabricated coatings was inspected by JSM-6490AL scanning electron microscope, SEM (JEOL, Tokyo, Japan), with 10 kV acceleration voltage. The samples were coated by a thin layer of gold (15 nm). For cross-section SEM imaging, the specimens were cryogenically fractured in liquid nitrogen, and then the surfaces of the fractures were coated by a thin layer of gold (15 nm) again. The morphology of the SCCNTs used in this study was analyzed by transmission electron microscopy, TEM, (JOEL JEM 1011 instrument with an acceleration voltage of 100 kV). A small amount of SCCNTs were dispersed in MIBK (0.05 wt % in solution) and from that a 20 μL quantity was dropped on copper grids (200 mesh), which was then dried under vacuum overnight.

The tensile measurements were conducted on five specimens for each composite type, according to ASTM D882 Standard Test Methods for Tensile Properties of Thin Plastic Sheeting, with an Instron dual column tabletop universal testing System 3365 with 50 mm·min^−1^ cross-head speed. Samples were cut with a standard dog-bone press. Dimensions of the test specimens were 25 mm × 4 mm.

The dynamic-mechanical thermal analysis (DMA) characteristics were measured on rectangular cut specimens (20 mm × 100 mm × 1 mm) in tensile mode under 10 Hz frequency using a TA Instruments Q800 machine. DMA spectra, namely storage modulus (E’) and mechanical loss factor (tanδ), were measured in the temperature range 170–290 K at a heating rate of 2 K/min. 

A special horizontal tensile tester was used for the measurement of electrical conductivity of the specimens (8 cm × 4 mm × 1 mm) during extensional and retraction mode of straining. The horizontal universal testing machine (model 1445) was coupled with a volume resistivity measurement set up for continuously reading the variation of resistivity of the sample during extension and retraction cycles. A constant crosshead speed was maintained to give a specified strain rate. Flexible copper foils were used to make electrode contacts with the rubber nanocomposites with electrode spacing of about 1/2 cm.

Thermal conductivity measurements were made with a TCi Thermal Conductivity Analyzer (Canada). The measurement method is based on modified transient plane source (ASTM D7984) with a transient line source that conforms to ASTM D5334, D5930, and IEEE 442-1981 standards. For each SCCNT concentration, at least four samples were measured, and averages were reported. Each sample was measured at least 10 times consecutively to ensure repeatability during the measurement as well. In order to have the best accuracy in measurements, six biocomposite papers of about 100 micron thick were stacked on top of each other to produce a half millimeter-thick composite by slightly compressing at 80 °C to enable self-adhesion. To reduce contact resistance, a drop of deionized water was deposited between the sensor and the samples, as indicated by the manufacturer.

## 3. Results

### 3.1. Rheological Characterization of the Dispersions 

Strain-dependent viscosity and storage and loss moduli (G’ and G’’) of pure rubber, rubber/lecithin, and SCCNT-dispersed rubber/lecithin solutions are displayed in Figure 3. Both rubber and rubber/lecithin blends were 33 wt % in the solvent MIBK. As seen in Figure 3a, viscosity of both pure rubber and rubber/lecithin solutions were shear rate-independent until about 500/s strain rates. No shear thinning was observed in these solutions. Afterwards, a small extent of shear thickening occurred that was more prominent in the case of natural rubber/lecithin solution. The shear thickening could be due to changes in the intramolecular to intermolecular interaction strength between rubber and protein molecules. In general, shear thickening occurs due to polymer segments that are capable of association into coil-like agglomerates instead of remaining in extended chain form [52], but shear thickening was not significant, particularly when compared to solutions containing SCCNTs (Figure 3a). Adding 10 wt % or 20 wt % SCCNTs into the rubber/lecithin solutions increases the viscosity significantly at low shear rates. Both solutions displayed a shear thinning behavior with slight increase in shear thinning after a shear rate of about 10/s. As can be seen in Figure 3a, the degree of shear thickening of both SCCNT suspensions after 500/s shear rate range was much stronger compared to only polymer/protein solutions. The sudden increase in viscosity can be associated with a dilatant fluid behavior that is most probably due to sudden increase in packing fraction of the nanoscale additives in suspension [53,54], which resembles discontinuous shear thickening. It has been demonstrated that when highly concentrated solutions containing nano or microscale particles are sheared, the particles try to go around each other but often cannot take a direct path, and as a result their packing volume expands (dilates) [54]. 

Figure 3b displays dynamic rheological response of rubber, rubber/lecithin, and SCCNT-dispersed rubber/lecithin suspensions. Both polymeric dispersions exhibited a liquid-like behavior with G’’ > G’ without the SCSNTs, with a practically linear viscoelastic behavior up to 100% strain. Both G’ and G’’ increased by adding SCCNTs. In the case of 10 wt % SCCNTs-suspended polymer/protein solutions, both elastic and loss moduli were linear up to 100% strain, similar to unfilled suspensions. This is known as the linear viscoelastic region (LVR). However, when the SCCNT concentration was increased to 20 wt %, LVR was lost after about 5% strain, as seen in Figure 3b. When the strain surpassed the critical value (around 100%), all solution G′ and G′′ values decreased immediately, while G’ remained less than G′′. A sharper decline was evident in the suspensions with 10 wt % SCCNTs, indicating more severe and potentially non-recoverable destruction of the fibril-like SCNCNT structures in the dispersions [55]. Note that the detailed rheological analysis of these suspensions is beyond the scope of this work, however, the suspensions with 20 wt % SCCNTs appear to have more stable elastic and loss moduli against high strain values (>100%) that might resist creep better under higher strain values. 

### 3.2. Morphological Features of the Conductors 

**Figure 4:**
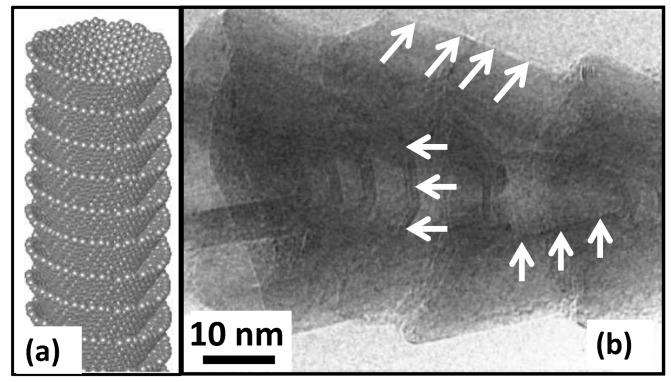
(**a**) An ideal representative stacked cup or cup-in-cup structure of a SCCNT and (**b**) high-resolution TEM image of coarse/irregular shaped stacked cup structure of the nanofibers used in the present study.

The SCCNTs utilized in this work were hollow, but the open tube diameter was not constant due to the cup-in-cup feature demonstrated schematically in Figure 4a. Figure 4b is the high-resolution TEM image showing the stacked-cup structure of the carbon nanofibers. White arrows indicate the graphitic edges of the cup-like structure and the open bottom part of the cups. The morphology of the SCCNTs, shown in Figure 4b, are rather coarse in the sense that the graphitic edges are much thicker than other SCCNTs reported in the literature [49] (see Figure 1). For this reason, they are generally referred to as hollow carbon nanofibers. 

**Figure 5:**
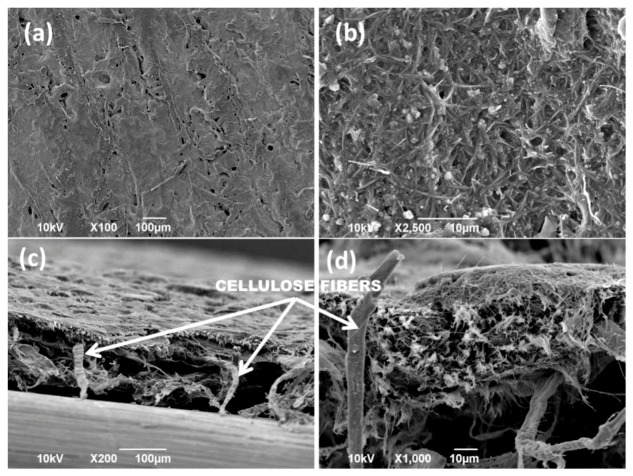
(**a**,**b**) Top surface SEM images of 20 wt % SCCNT containing biocomposite flexible papers impregnated into cellulosic tissues by hydraulic pressing. Images (**c**,**d**) show the cross-section of the paper obtained by breaking the liquid nitrogen frozen paper into two pieces.

Figure 5 demonstrates the SEM morphological features of the conductive paper fabricated by using the 20 wt % SCCNT rubber/lecithin solutions. The morphology was obtained by brush-painting both sides of the cellulosic tissues and subsequent drying. Afterwards, the biocomposite was compacted by using a hydraulic press at 20 tons for 2 min under ambient conditions. The surface micro-morphology of the biocomposite is shown in Figure 5a,b. Close inspection of Figure 5b indicates random but well-dispersed SCCNTs resembling a collection of fishbone-like zones. The cross-section SEM images of Figure 5c,d clearly show that the cellulose fiber network has been completely coated and impregnated with a SCCNT biocomposite forest. The structure resembles a composite textile with a dual-scale fiber network, such as a nanofiber network, embedded in a microfiber network [56]. Maintaining such a structure will be a key electrical and thermal conductivity performance criterion against various bending and folding tests that will be discussed next.

### 3.3. Mechnical Properties of the Composites 

**Figure 6:**
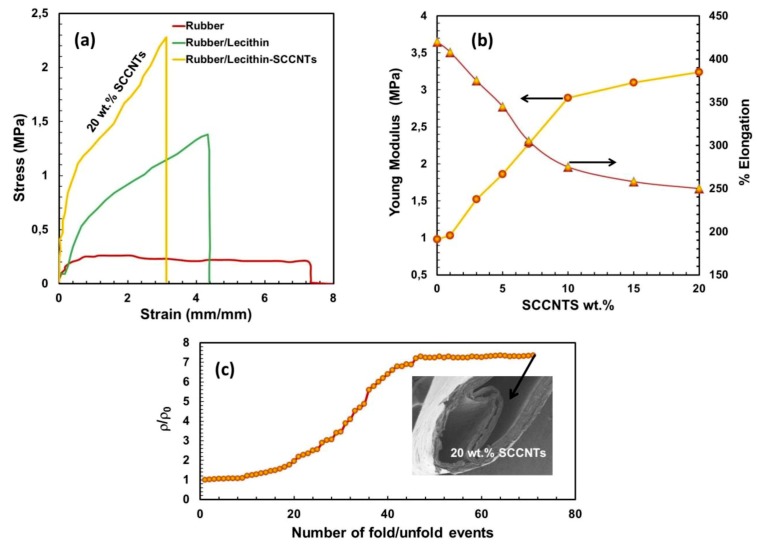
(**a**) Stress-strain behavior pure rubber and some select bio-nanocomposites before impregnating into the cellulose membrane. As before, rubber/lecithin blend (matrix) is always maintained at 1:1 ratio. (**b**) Changes in elastic modulus and elongation at break values as a function of SCCNT concentration in the rubber/lecithin matrices. (**c**) Folding/unfolding resistance of the 20 wt % SCCNT-loaded bio-nanocomposite after impregnating into the cellulose fiber network (membrane).

Before impregnating into the cellulosic network, the mechanical properties of the rubber and rubber/lecithin matrix filled with different concentrations of the SCCNTs were investigated. Figure 6a shows the stress-strain behavior of selected samples. Pure rubber is highly elastomeric, with an elongation at break value of about 740%. Adding 50 wt % lecithin reduces elongation at break value to about 420%. In fact, this is the matrix in which SCCNTs were compounded. When 20 wt % SCCNTs were added, the stress-strain curve significantly changes, as seen in Figure 6a. Elongation at break value drops down to about 250%. More explicitly, the effect of the intermediate SCCNT concentrations on Young’s moduli and the elongation at break values of the rubber/lecithin blends are detailed in Figure 6b. The most conductive biocomposite has a Young’s modulus of about 3.4 MPa compared to the unfilled rubber/lecithin composite, which has about 1 MPa of elastic modulus. As can be seen in these results, the most conductive rubber/lecithin blend is still relatively soft and for this reason was chosen to be impregnated into the cellulosic fiber network for utilization like a conductive paper. 

Finally, the folding/unfolding resistance of the 20 wt % SCCNT-loaded rubber-lecithin biocomposite papers was tested by measuring the resistivity at the end of each unfolding after the biocomposite was folded over like a paper while running a 0.5 kg weight over the fold line four consecutive times. The results are displayed in Figure 6c. For up to about 20 folding/unfolding events, the relative resistivity (ρ/ρ_0_, does not change much, remaining close to 1. However, after this point, a steady increase in relative resistivity (decrease in conductivity across the fold line) starts until about 45 folding/unfolding cycles are reached. From this point onward, ρ/ρ_0_ remains at about 7 and does not change until the end of 70 cycles, after which formation of some cracks and partial delamination of layers were noticed, and the experiments were terminated. Nonetheless, the biocomposite conductors resisted such severe (pressing over fold line with weight) folding/unfolding events for up to 70 cycles without losing conductivity even by an order of magnitude.

### 3.4. Potential Chemical Interactions

**Figure 7:**
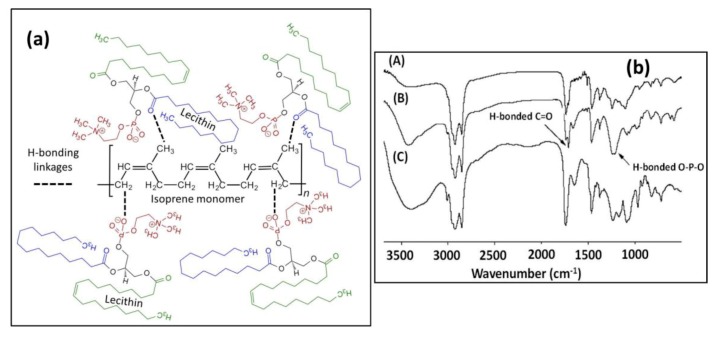
(**a**) Schematic hydrogen bonding interactions between natural rubber isoprene structure and lecithin, and (**b**) FTIR results of rubber/lecithin blends (1:1) cast from different solvents, indicating potential H-bonding groups. (**A**) Solvent: Heptane, (**B**) solvent: MIBK and spectrum (**C**) solvent: Toluene.

It is expected that solution blending of soy lecithin and natural rubber will not form a new compound but will simply be a blend. However, the protein and the rubber can interact with each other through certain electrostatic, hydrogen, or Van der Waals forces. Infrared spectroscopy (FTIR) has been used to identify if there was any hydrogen bonding between lecithin and the natural rubber latex when the two components were blended. It is known that intermolecular interactions caused by hydrogen bonding can be detected by FTIR, since the vibrational modes or characteristics of the interacting acceptor/donor moieties change if hydrogen bonding is established. Hydrogen bonding via oxygen atoms leads to a decrease in vibrational force constants and diminishing trends in intensities of frequency. In addition, many lipid compounds or fatty proteins like lecithin contain amine and/or amide groups as well as O−P−O bonding in their structure. For instance, it has been reported that the asymmetric O−P−O vibrational band is very sensitive to hydrogen bonding or electrostatic interactions because of the shift of its frequency from 1250 cm^−1^ for non-interacting to 1230 cm^−1^ in H-bonded state [57]. Similarly, FTIR bands pertaining to amine or amide groups shift upon establishing H-bonding [58]. The chemical structure of soy lecithin, natural rubber, and the proposed hydrogen bonding interactions are shown in Figure 7a. 

In Figure 7b, we characterized three natural rubber/lecithin blends (1:1) prepared in different ways in order to understand if the potential interactions depend on the blending process or the solvent used. Spectrum (A) in Figure 7b was made by blending both compounds in heptane and casting and drying films in a similar way with MIBK. Spectrum (B) is from MIBK and spectrum (C) is from toluene. Both toluene and heptane are non-polar solvents, whereas MIBK is a polar solvent. It can be seen in Figure 7b that both blends prepared in non-polar solvents gave FTIR spectra similar to that obtained from MIBK (spectrum B). This indicates that there exist both free and interacting phospholipids due to soy lecithin. It is interesting to note that a small band appeared at 1713 cm^−1^ in blends obtained from both heptane and MIBK dispersions. This minor band indicates hydrogen bonding via C=O (see Figure 7a). Looking at the relative intensity of this band in heptane and toluene (almost none) indicates that hydrogen bonding via C=O may be decomposed by these solvents. Other hydrogen bonding sites between the rubber and the protein were confirmed by the O−P−O stretching band. The O−P−O asymmetric stretching at 1219 and 1240 cm^−1^ was clearly visible in the case of MIBK, whereas it was absent in both blends obtained from toluene and heptane. Hence, we argue that soy lecithin can aggregate in MIBK in such a way that it can establish links with the rubber (isoprene) chains via hydrogen bonding through both phosphate and carboxyl groups. The fact that such interactions are not very clearly observed in other solvents could be due to the fact that most phospholipids are complex molecular structures and form solvent-dependent particular micelles and reverse micelles structures that might inhibit or favor molecular interactions with other polymers co-suspended in those solvents [59,60].

### 3.5. Thermomechnical Properties and Electrical Reistivity under Deformation 

**Figure 8:**
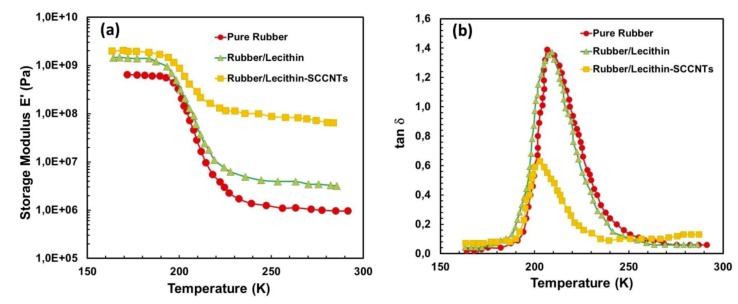
Dynamic-mechanical thermal analysis (DMA) measurements of some select samples. (**a**) Storage modulus as a function of temperature for pure rubber, rubber/lecithin blend (1:1) and 20 wt % SCCNT-loaded bio-nanocomposite (no cellulose impregnation) and (**b**) loss factor (tanδ) of the same materials as a function of temperature.

Figure 8 shows the change in storage modulus (E’) (Figure 8a) and the loss factor tanδ (Figure 8b) at 10 Hz as a function of temperature (170–290 K) for the rubber/lecithin blends, including pure rubber. At low temperatures, the rubber is in the glassy state with a modulus of about 0.6 GPa. With increasing temperature, the modulus rapidly declines by 3 orders of magnitude corresponding to the glass–rubber transition. This drop in modulus is ascribed to energy dissipation related to compliant motions of long chain sequences. This physical aspect is also observed in the corresponding relaxation process, where the loss factor tanδ passes through a maximum at 210 K (−63.15 °C). Similarly, the rubber/lecithin (1:1) blend demonstrates a glassy state at low temperatures but with a higher storage modulus of about 1.1 GPa, and the loss factor, tanδ, also peaks at about 208 K. When 20 wt % SCCNTs are added, however, the temperature corresponding to the maximum tanδ decreases to about 201 K. A similar effect was also reported in cellulose whisker-reinforced natural rubber composites [61]. This inverse trend is usually observed in reinforced composites and is interpreted as a decrease of chain mobility (or equivalently, an increase of Tg) due to interaction with nanoscale fillers. Moreover, the storage modulus in Figure 8a declines by about an order of magnitude in the presence of SCCNTs compared to pure rubber or rubber/protein composites. 

Below the glass transition temperature (Tg), addition of SCCNTs to the rubber/lecithin blend does not significantly increase the storage modulus, as seen in Figure 8a, but above the glass-rubber transition point, addition of SCCNTs significantly increases the storage modulus, indicating a homogeneous dispersion of SCCNTs in the polymer/protein matrix once the composite is maintained at a rubbery state. Although fully detailed dynamic mechanical properties of the composites are beyond the scope of this work, the results indicate that both lecithin and SCCNTs endow higher strength to pure natural rubber.

**Figure 9:**
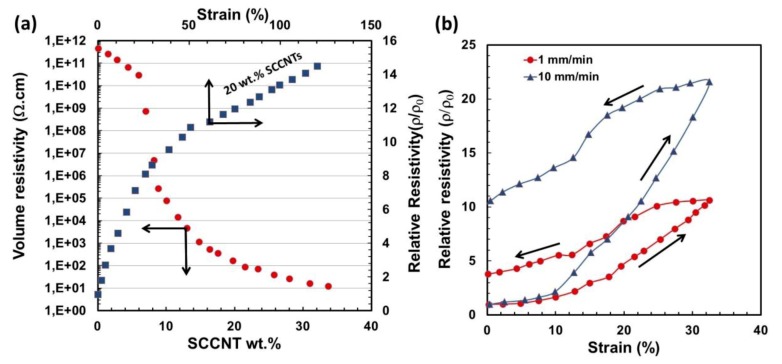
(**a**) Volume resistivity as a function of SCCNT concentration and relative resistivity changes as a function of percent strain and (**b**) effect of stain rate on the relative resistivity for 20 wt % SCCNT containing biocomposite. All data were acquired after impregnation into the cellulose network.

Next, we investigate the response of electrical resistance of the biocomposites to strain. Figure 9a shows the changes in electrical volume resistivity (ρ) of the composites as a function of SCCNT weight percent as well as changes in the relative resistivity (instantaneous resistivity under strain divided by the initial resistivity; ρ/ρ_0_) as a function of applied strain (0.5 mm/min) for the 20 wt % SCCNT biocomposite. The percolation occurs at about 5 wt % SCCNT concentration, and the volume resistivity at 20 wt % SCCNT loading was measured to be about 85 Ω·cm. When this composite was subjected to strain, relative resistivity increased sharply to 8 at about 5% strain and continued to increase with a lower trending slope and reached about 13 at 100% strain, an order of magnitude increase (decrease in conductivity). After about 120% strain, the conductive papers started to break apart and experiments were terminated.

Sensitivity of the relative resistivity (ρ/ρ_0_) of the conductive papers (20 wt % SCCNT) to strain rate is shown in Figure 9b. Stretching the biocomposites at 1 mm/min practically reproduces the relative resistivity value in Figure 9a at 32% obtained by a strain rate of 0.5 mm/min. Upon relaxing, however, (ρ/ρ_0_) settles at a higher value of around 4 at 0% strain. Stretching the sample with a faster rate (10 mm/min), however, reduces electrical conductivity further, and at 32% final strain point, (ρ/ρ_0_) value becomes 21. Upon relaxing back to zero at the same strain rate, (ρ/ρ_0_) rests at about 10.5 instead of 1 at 0% strain. These results based on 32% maximum strain or stretching could be acceptable and comparable to conducting textiles (wool, nylon, Lycra) made with single wall carbon nanotubes (SWCNTs) even though we utilized a cellulose support, which is much less stretchable than most textiles [62]. Mechanical response of such nano-carbon-based papers, such as cyclic response, is very important to maintain longer life cycles with stable electrical performance [63]. We did not conduct repeated elongation cycles, since conductive papers are generally not suitable for multi-step high elongation levels, although the rubber itself can be suitable as a standalone matrix. For the experimental conditions demonstrated in Figure 9, the samples recovered about 94% of their initial resistivity after 24 h in an oven at 70 °C. 

### 3.6. Thermal Conductivity 

**Figure 10:**
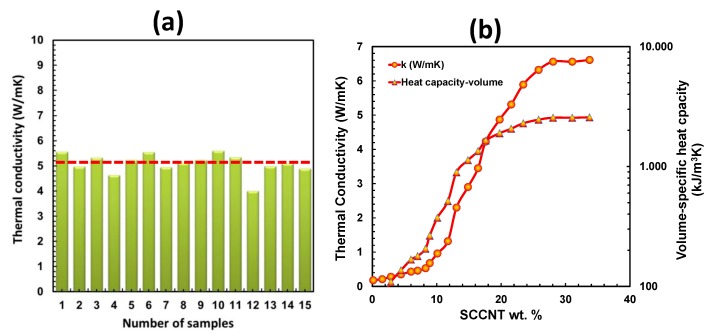
(**a**) Thermal conductivity of 15 different samples of 20 wt % SCCNT-containing biocomposite with an average value of 5.1 W/mK and (**b**) changes in thermal conductivity and volume-specific heat capacity of the biocomposites as a function of SCCNT concentration.

Through-plane thermal conductivity of the SCCNT biocomposites was measured using a commercial thermal conductivity meter employing transient plane source technique [64]. For the most conductive biocomposite paper with 20 wt % SCCNTs, 15 different samples were measured, and the average thermal conductivity was calculated to be 5.10 W/mK, as shown in Figure 10a. Figure 10b demonstrates measured thermal conductivity and thermal effusivity of biocomposites with varying SCCNT concentrations and calculated volumetric heat capacity (kJ/m^3^K) based on the relationship $e={\left(kΡC_{p}\right)}^{1/2}$, where *e* is thermal effusivity, *k* is thermal conductivity, *P* is density, and *C_p_* is specific heat capacity. As can be seen in Figure 10b, the percolation in thermal conductivity occurs much later than the electrical conductivity at about 12 wt % SCCNT compared to 5 wt % SCCNT concentration. This confirms various previously published works that demonstrated relatively low percolation thresholds for carbon nanotube composites indicated by sharp increases in electrical conductivity at very low CNT concentrations. On the other hand, thermal conductivity measurements on identical conductors showed no such early percolation threshold. Such a contrasting behavior is intriguing, since both transport processes are described by the same continuum equation [63]. The most plausible explanation supported by theoretical calculations indicates that higher CNT loadings in polymer composites intensely increase thermal contact resistance, which is directly reflected as lower thermal conductivity measurements [65,66].

Volumetric heat capacity (VHC) or volume-specific heat capacity is defined as the ability of a given volume of material to store internal energy while undergoing a specified temperature change but without experiencing phase changes. VCH is particularly important if such materials are to be used in smart thermal energy management systems, such as large-scale electronic cooling or buildings [67,68]. In general, high VCH materials with high thermal conductivity are required for heat storage applications, such as internal walls of a building, whereas low *k* and low VCH materials may be suitable for controlled thermal insulation [66]. The biocomposites developed in this work can serve both purposes, depending on the SCCNT concentration in the rubber-lecithin matrix, as shown in Figure 10b. For instance, the composite with 20 wt % SCCNT has a thermal conductivity of 5.1 W/mK with a VCH value of about 2000 kJ/m^3^K. They can also be suitable candidates as thermal interface materials [69].

## 4. Conclusions

Irregularly shaped cup-in-cup carbon nanotubes or stacked-cup carbon nanotubes (SCCNT) are, in fact, known as carbon nanofibers (CNFs). They are economically less expensive than standard CNTs. In this work, we used commercially available SCCNTs or CNFs to transform natural rubber-lecithin blends into conductive soft biocomposites using a benchtop high-pressure homogenizer. Natural polymers and the SCCNTs were dispersed in a green solvent known as methyl isobutyl ketone (MIBK). The resultant inks could be brush-on painted over cellulose fiber networks, and they were pressed into the texture by a hydraulic press. As such, we obtained flexible conductive papers with a volume resistivity of about 85 Ω·cm at 20 wt % SCCNT loading. The conductors had good resistance against severe folding (weight-pressed) and bending tests and did not undergo order-of-magnitude conductivity loss after 70 weight-pressed folding/unfolding cycles. The selected solvent enabled establishment of hydrogen bonding between natural rubber and soy lecithin. Thermomechanical measurements showed that the biocomposites have good stability below and above glass transition temperature points. Conducting biocomposites also had high through-plane thermal conductivity of 5 W/mK with a volumetric heat capacity of 2000 kJ/m^3^K, ideal for thermal interface heat transfer applications.
